# Recurrence patterns of locally advanced head and neck squamous cell carcinoma after 3D conformal (chemo)-radiotherapy

**DOI:** 10.1186/1748-717X-6-54

**Published:** 2011-05-24

**Authors:** Didem C Oksuz, Robin J Prestwich, Brendan Carey, Stuart Wilson, Mustafa S Senocak, Ananya Choudhury, Karen Dyker, Catherine Coyle, Mehmet Sen

**Affiliations:** 1 St James's Institute of Oncology, Leeds, UK; 2 Istanbul University, Cerrahpasa Medical Faculty, Department of Biostatistic, Istanbul, Turkey; 3 Christie Hospital, Manchester, UK

## Abstract

**Background:**

To establish recurrence patterns among locally advanced head and neck non-nasopharyngeal squamous cell carcinoma (HNSCC) patients treated with radical (chemo-) radiotherapy and to correlate the sites of loco-regional recurrence with radiotherapy doses and target volumes

**Method:**

151 locally advanced HNSCC patients were treated between 2004-2005 using radical three-dimensional conformal radiotherapy. Patients with prior surgery to the primary tumour site were excluded. The sites of locoregional relapses were correlated with radiotherapy plans by the radiologist and a planning dosimetrist.

**Results:**

Median age was 59 years (range:34-89). 35 patients had stage III disease, 116 patients had stage IV A/B. 36 patients were treated with radiotherapy alone, 42 with induction chemotherapy, 63 with induction and concomitant chemoradiotherapy and 10 concomitant chemoradiotherapy. Median follow-up was 38 months (range 3-62). 3-year cause specific survival was 66.8%. 125 of 151 (82.8%) achieved a complete response to treatment. Amongst these 125 there were 20 local-regional recurrence, comprising 8 local, 5 regional and 7 simultaneous local and regional; synchronous distant metastases occurred in 7 of the 20. 9 patients developed distant metastases in the absence of locoregional failure. For the 14 local recurrences with planning data available, 12 were in-field, 1 was marginal, and 1 was out-of-field. Of the 11 regional failures with planning data available, 7 were in-field, 1 was marginal and 3 were out-of-field recurrences.

**Conclusion:**

The majority of failures following non-surgical treatment for locally advanced HNSCC were loco-regional, within the radiotherapy target volume. Improving locoregional control remains a high priority.

## Introduction

Head-and-neck squamous cell carcinoma (HNSCC) is the sixth most common malignancy worldwide, responsible for approximately half a million new cases every year [1]. Approximately 60% of patients with HNSCC present with locally advanced, but non-metastatic disease (stage-III or IVA/B) at diagnosis. Based upon organ preservation studies [2,3], radiotherapy is an accepted alternative to surgery. The results of radical radiotherapy regimens have been further improved by the use of induction chemotherapy [4], concurrent chemoradiotherapy [4], and concurrent epidermal growth factor inhibitors [5]. In parallel, radiotherapy techniques have developed rapidly; conformal radiotherapy (CRT), accelerated schedules [6] and intensity modulated radiotherapy (IMRT) [7] have been used to improve the therapeutic ratio between tumour control and normal tissue toxicity.

Historically, locoregional failure has been the predominant pattern of relapse following non-surgical treatment [8]. With the rapid advancement of non-surgical treatment strategies, it is critical to document the pattern of treatment failure in relation to, the radiotherapy dose distributions. These data are required to guide whether future improvements should be focused on improving local and/or regional control or, on reducing the development of distant metastases (DM). The former may involve modifications in target volume definition, delivery technique, or dose escalation. However, if DM is an increasing problem, consideration could be given to prioritizing the delivery of systemically active therapy. Therefore, the aim of this retrospective study is to determine recurrence patterns among HNSCC patients treated with radical three-dimensional (3D) CRT with or without chemotherapy, and to correlate the sites of local-regional recurrence (LRR) to previously treated radiotherapy fields and dose distribution.

## Materials and methods

After institutional review board approval, we retrospectively reviewed the medical records of patients with locally advanced stage III/IV HNSCC treated with 3D-CRT with curative intent at the Yorkshire Cancer Centre between January 2004 and December 2005. Patients with nasopharynx carcinomas were excluded. Patients who had undergone initial therapeutic surgery to the primary tumour site were excluded.

### Pre-treatment work up

Diagnostic staging routinely consisted of physical examination, nasoendoscopy, computed tomography (CT) or magnetic resonance imaging (MRI) scans of the head and neck, CT of thorax, direct endoscopy under anaesthesia and histological confirmation.

### Radiotherapy treatment planning

The patients were treated supine, immobilised with a beam directional perspex shell. CT images for treatment planning were obtained at 2-5 mm intervals from the vertex to below the carina. The CT data was loaded into the Helax-TMS VG-1B treatment planning system. One of two methods was routinely used for target volume definition. The first of these was utilised for patients who were to be treated using a parallel opposed pair to the high dose region; a planning target volume (PTV) was directly defined using virtual simulation. The need to outline a gross tumour volume (GTV) and clinical target volume (CTV) to aid definition of the PTV was at the discretion of the clinician. The second method of outlining the target volume was used for patients who were not intended to be treated with parallel opposed pair. A GTV was outlined as primary tumour and clinically and/or radiologically involved lymph nodes. A CTV was created to include areas and lymph nodes at high risk of tumour involvement; this was by auto-expanding the GTV by 10-20 mm, individualising this to exclude areas of air or bone without evidence of tumour invasion, and expanding the CTV to include high risk nodal areas. In general, bilateral level II, III, IV and V lymph nodes were included within the CTV with some exceptions relating to patients' co-morbidities. Level IB and retropharyngeal lymph nodes were variably included depending on tumour site and stage. The CTV was subsequently expanded automatically in 3D by 3-5 mm to create a PTV.

Organs at risk were routinely outlined on the planning CT images. For conventionally fractionated treatment schedules (2Gy per fraction) a maximum dose of 46Gy to spinal cord, 54Gy to brainstem was accepted. For the accelerated hypofractionated schedule of 55Gy in 20 fractions, a maximum dose of 40Gy to spinal cord, 42Gy to brainstem was accepted.

Radiotherapy was with 6 MV photons ± posterior electron fields. Treatment was commonly planned using a 2 phase technique of lateral opposed pair of multiple field-in-fields, with the posterior border moved anterior to spinal cord prior to reaching spinal cord tolerance and matched posterior electron fields used to treat nodal areas overlying the cord. A 6 MV photon anterior neck field was matched geometrically to the lateral opposed photon fields. Alternatively, following definition of contoured target volumes, treatment was with a single phase conformal 5-7 field plan. Treatment was planned to provide adequate coverage of the primary target and lymph nodes at risk according to ICRU-62 guidelines [9].

During the period of the study, different radical radiotherapy regimes were in standard use. The most commonly used schedules were conventionally fractionated 66-70Gy in 33-35 fractions, and an accelerated hypofractionated schedule of 55Gy in 20 fractions, prescribed to the 100% isodose within the PTV. The choice of conventionally or hypofractionated radiotherapy reflected historical practice and clinician preference. Using the lateral opposed pair technique, an anterior neck field of 50Gy in 25 fractions for the conventionally fractionated regimen or 40Gy in 15 for the hypofractionated regimen, was used. All patients were treated once daily, five times a week.

### Chemotherapy

The use of chemotherapy was based upon clinicians' assessment of multiple factors, including age, co-morbidity, performance status, tumour extent and social support. For patients who were treated with induction chemotherapy, chemotherapy was administered as 2-3 cycles of cisplatin 80 mg/m^2 ^Day1 and 5-fluorouracil 800 mg/m^2 ^Days 2-5, every three weeks. For patients treated with concomitant chemotherapy, cisplatin 100 mg/m^2 ^was delivered up to three times for conventionally fractionated schedules at 3 week intervals, and at a dose of 80 mg/m^2 ^on day 1 and 28 of the four week hypofractionated radiotherapy schedule.

### Analysis of response to treatment and follow-up

Tumour response was assessed 4 months after the completion of the treatment. Evaluation of tumour response consisted of clinical examination, nasopharyngolaryngoscopy and CT or MRI imaging of the primary site and the neck. Any suspicious findings in the primary tumour site or neck were evaluated by biopsy. Patients with less than a complete response (CR) were evaluated for salvage surgery. All patients were routinely followed up with physical examination and flexible endoscopy every 6-8 weeks in the first year after treatment, every 3 months for an additional 2 years, every 6 months years 4 and 5.

### Definition of failure site

**Figure 1:**
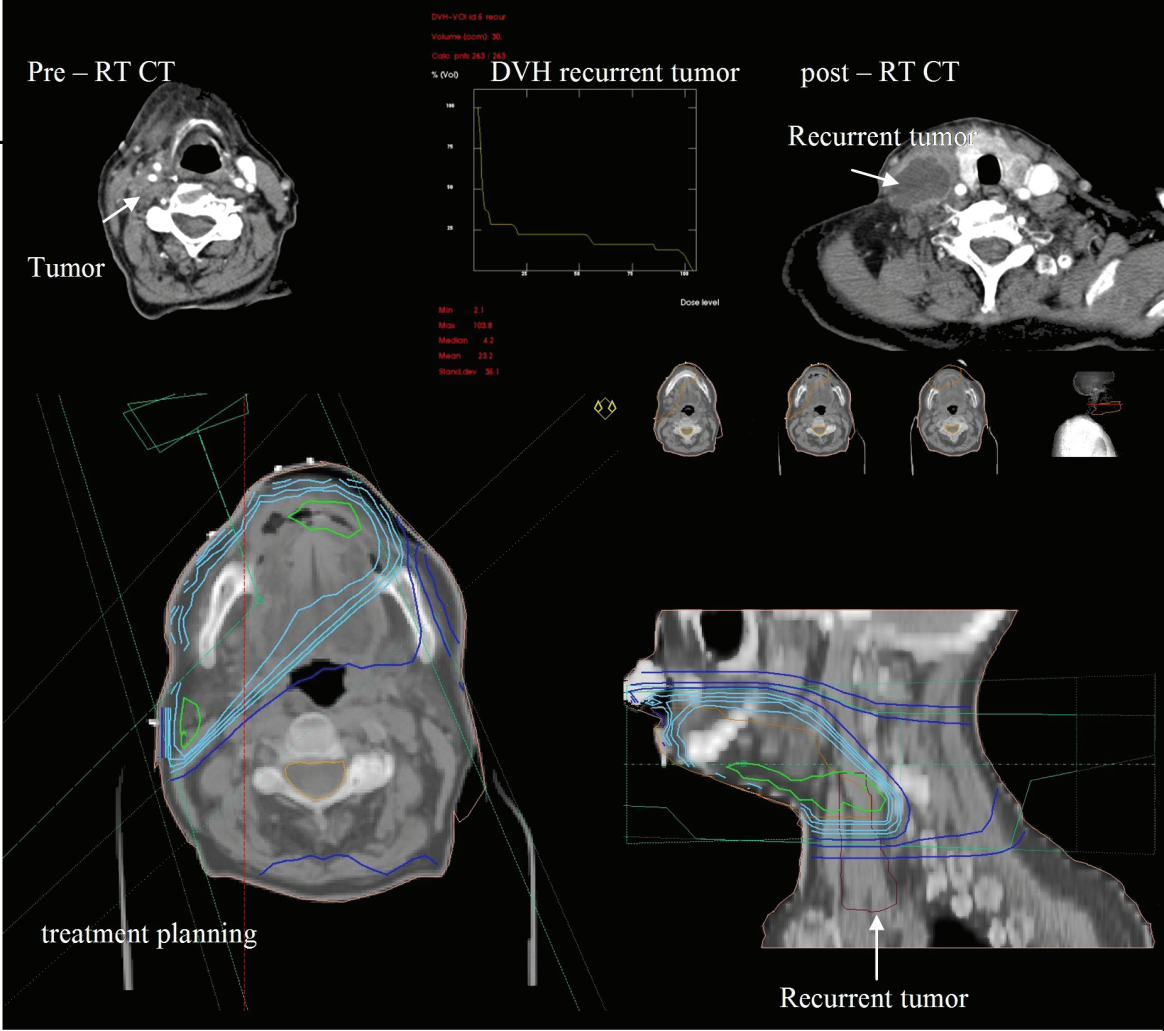
**Reconstruction of site of tumour recurrence on pretreatment planning CT and DVH analysis to determine the site of recurrence in relationship to treatment volume: An example of out-of-field recurrent tumour in a patient with T2N1 oral cavity tumor(patient-5)**.

**Figure 2:**
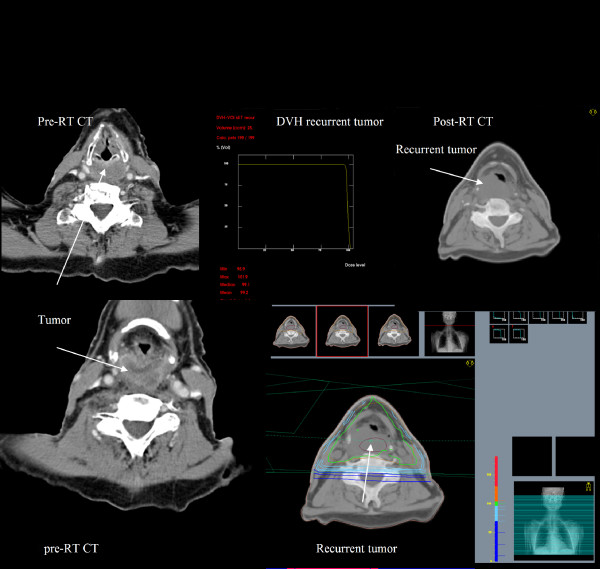
**Reconstruction of site of tumour recurrence on pretreatment planning CT and DVH analysis to determine the site of recurrence in relationship to treatment volume: An example of infield recurrent tumour in a patient with T4N0 hypopharynx tumor(patient-14)**.

Each local or regional treatment failure site was confirmed pathologically and reviewed within the multidisciplinary head-and-neck team. As part of the study, the imaging of the all patients who experienced LRR was reviewed by a head and neck specialist diagnostic radiologist, to identify the precise site of LRR. The radiologist, using information on the imaging demonstrating recurrence, including size of recurrence and relationship to anatomical structures, reconstructed the recurrent volume of tumour (Vrecur) by contouring, on the original planning CT images. The original treatment plan was applied, and dose volume histograms (DVH) were obtained for the reconstructed recurrent tumour. The local and regional recurrences were classified as "in-field," in which 95% or more of the Vrecur was within the 95% isodose based upon DVH assessment; "marginal," in which 20% to 94% of Vrecur was within the 95% isodose; or "out-of-field," in which less than 20% of Vrecur was within the 95% isodose line (Figure 1,2). Recurrences were defined as local if they were within the zone of the primary tumour, and as regional if they occurred elsewhere including neck lymph nodes.

### Statistical analysis

All statistical computations were performed using PASW-18. Variables were compared using student-t, Mann-Whitney-U or chi-square test according to the variable properties. Kaplan-Meier analysis with a log-rank test was used for survival analysis. The Cox proportional hazards regression model was used for multivariate survival analyses. Survival was calculated from the date of diagnosis. Locoregional control (LRC) time was defined as the time from the date of diagnosis to date of local or regional relapse. For distant metastases free survival (DMFS), first recurrence at distant site was taken as an event. Salvage of recurrences was not included in the evaluation of LRC or progression-free survival (PFS). For the PFS analysis, progression was defined as locoregional progression or distant progression. Cause-specific survival (CSS) events were defined as death from cancer or treatment complications.

## Results

### Patient and tumour characteristics

A total of 151 patients with locally advanced non-nasopharyngeal HNSCC were treated at the Yorkshire Cancer Centre between January 2004 and December 2005 using 3D-CRT with or without chemotherapy. 7 patients had undergone prior unilateral neck dissections. One hundred and nine (72%) were male. Median age was 59 years (range: 34-89 years).

Tumour and treatment characteristics are summarized in Table 1. The primary tumour site was the oropharynx in 81 patients (54%), the larynx in 32 (21%), the hypopharynx in 21 (14%), the oral cavity in 14 (9%). All patients had histologically confirmed squamous cell carcinoma. Histological grading was recorded in 134 patients and 76 (50.3%) had poorly differentiated squamous cell carcinoma. Stage distribution was classified according to the 2002 TNM staging system of American Joint Committee on Cancer (AJCC). Thirty-five (23%) patients had stage III disease while 116 (77%) patients had stage IV A/B (Table 2).

**Table 1:** Tumour and treatment characteristics for all patients

	N	%
**Primary tumour site**		

Oropharynx	81	53.6

Larynx	32	21.2

Hypopharynx	21	13.9

Oral cavity	14	9.3

Unknown primary	3	2

**Histological grade of squamous cell carcinoma**		

Well differentiated	6	4

Moderately differentiated	52	34.4

Poorly differentiated	76	50.3

Not recorded	17	11.3

**Overall stage (AJCC)**		

III	35	23.2

IVA	87	57.6

IVB	29	19.2

**Treatment**		

Radical radiotherapy alone	36	23.8

Chemoradiotherapy (chemoXRT)	115	

Induction chemo + XRT	42	27.9

Induction chemo + concomitant chemoXRT	63	41.7

Concomitant chemoXRT	10	6.6

Abbrevations: Chemo: chemotherapy; XRT: radiotherapy

**Table 2:** Detailed distribution of primary tumour and nodal stages

	N0	N1	N2	N3	Total
T1	-	4	10	3	17
T2	-	12	15	5	32
T3	12	7	13	6	38
T4	18	7	28	8	61
TX	-	-	3	-	3
**Total**	30	30	69	22	151

### Treatment details

One hundred and fifteen (76.2%) patients were treated with a combination of chemotherapy and radiotherapy. Of these 115 patients, 42 (36.5%) patients were treated with induction chemotherapy followed by radiotherapy, 63 (54.7%) patients with induction chemotherapy and concomitant chemoradiotherapy and 10 patients (8.7%) with concomitant chemoradiotherapy. Thirty-four of 73 patients received 2 or more cycles. The remaining 36 patients were treated with radiotherapy alone (Table 1).

The majority of patients were treated with hypofractionated radiotherapy. 80 patients received 55 Gy in 20 fractions and 14 patients received other hypofractionated regimes (i.e. 3 patients with 60 Gy in 25 fractions or 11 patients with 65 Gy in 30 fractions). 50 patients were treated with conventional radiotherapy (i.e. median 68 Gy (range: 66-70 Gy) in once daily fractions of 2 Gy each). Seven of 151 (4.6%) patients did not receive the planned radiotherapy dose. In five cases this was due to acute treatment toxicity necessitating a reduced total dose, one patient had disease progression during treatment and one patient suffered colonic perforation.

### Response to treatment

After induction chemotherapy 84/105 patients (80%) had a clinical CR or PR. One hundred and seventeen of 151 (77.5%) patients had CR 4 months after their radical treatment. Patients with a PR (14.6%), stable (0.7%) or progressive (2%) disease were evaluated for salvage treatment. Eight patients underwent salvage surgery (5 neck dissections and 3 laryngectomies ± neck dissections) for residual disease achieving complete macroscopic tumour clearance. Tumour response was not assessed in 8 patients (5.3%) because of death from toxicity (6 patients) or unrelated causes (2 patients). Thus, including salvage surgery, 125 of 151 (82.8%) patients achieved a CR to treatment.

### Survival rates

The median follow-up of all patients was 38 months (range:3-62 months). Forty four (29.1%) patients died of cancer, 17 (11.3%) of intercurrent disease. Treatment related deaths occurred in 6 (4%) patients with 5 dying of aspiration pneumonia. Three out of 6 died after the completion of 55Gy in 20 fractions over 4 weeks. For the entire cohort of 151 patients the OS rates were 67.5% and 58.3%; the CSS rates were 75.1% and 66.8% and PFS rates were 72.5% and 67.2% at 2 and 3-years respectively.

### Patterns of recurrence of patients who had CR after the treatment

**Figure 3:**
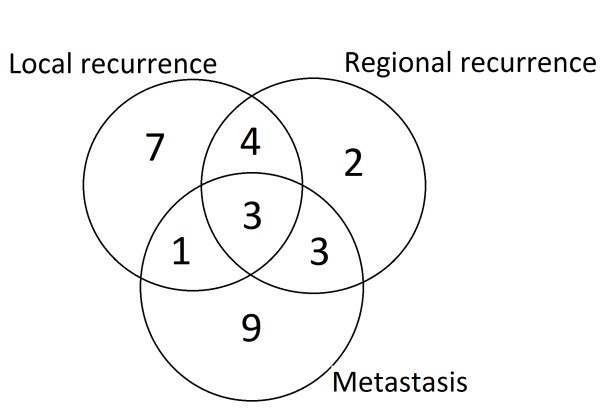
**The failure patterns of 125 patients who had complete response after the treatment**.

Median follow up for the 125 who achieved a CR to treatment, (including the 8 patients who underwent salvage surgery) was 40 months (range:6-62 months). The patterns of subsequent failure in these patients are shown in Figure 3. Disease recurred in total of 29 of the 125 patients. The median time to failure of these 29 patients was 18 months (range:7-38 months). Seven patients developed isolated local recurrence with a median time to recurrence of 16 months (range:13-23 months). One of these patients underwent a salvage laryngectomy, and has subsequently remained disease free. A combination of local and regional recurrence (in the absence of DM) occurred in 4 patients after median 13.5 months (range:12-17 months) and 3 of these underwent salvage surgery. Two patients had an isolated regional recurrence, occurring at 13 and 22 months; one patient was treated with palliative chemotherapy and the other with best supportive care alone. Hence overall, a total of 20 out of 125 patients experienced local and/or regional failure with a median time to failure of 16.5 months (range:11-26 months). Among the complete responders, 3-year local and regional control rates were 86.8% and 89.5%, respectively. A total of 16 of 125 patients with CR developed DM after a median of 22.5 months (range 7-38 months). The 3-year DMFS rate in patients with CR was 87.3%.

The details of patients and tumours in cases, where LRR were noted, are provided in Table 3. Radiotherapy planning CT images were available for 19 of the 20 patients. For the 14 local recurrences with planning data available, 12 recurrences were in-field, 1 was marginal, and 1 was out-of-field. Of the 11 regional failures with planning data available, 6 were in-field, 1 was marginal, 3 were out-of-field recurrences, and one patient experienced both infield (right side) and outfield (left side) recurrences. Patients with laryngeal cancer had the most LRR; they accounted for 50% of all recurrences although they only comprised 21.2% of all tumours. Twelve of 20 LRR patients received 55 Gy in 20 fractions of radiotherapy, and 9 of these patients experienced in-field recurrences.

**Table 3:** Details of patients who developed a local or/and regional failure

Pt. no	Primary tumor site	Stage	XRT dose (Gy/fr)	Type of relapse	Site of first failure	Time to failure (mo)	Type of failure	Metastasis
**Primary Radical radiotherapy**								

1.	Hypopharynx	T3N0	55/20fr	L	Right Hypopharynx	23	infield	-

2.	Larynx (Glottic)	T4AN0	55/20fr	L, R	Anterior Ventricle Level VI LN	17	Infield (L) Outfield (R)	-

3.	Oropharynx	T1N2A	65/30fr	R	Right Level III LN Left Level II, III, IV LN	21	Infield (Right side) Outfield (Left side)	Lung (Synchronous)

4.	Larynx (Supraglottic)	T3N0	55/20fr	R	Left Level II-III LN	11	Outfield	Lung, bone (Synchronous)

5.	Oral cavity	T2N1	55/20fr	R	Right Level IV-V-VI	13	Outfield	-

6.	Larynx (Glottic)	T3N0	55/20fr	L, R	Glottic region Level II-III LN	12	NA	-

7.	Oral cavity	T4AN1	55/20fr	L	Left mandibular alveolus	14	Infield	-

8.	Larynx (Glottic)	T4AN0	55/20fr	L	Stoma	26	Infield	Lung (Synchronous)

**Concomitant chemoradiotherapy**								

9.	Larynx (supraglottic)	T1N2B	70/35fr	L, R	Base of tongue Tumor nodules around stoma and skin	14 (L) 16 (R)	Infield	-

**Induction chemotherapy + radiotherapy**								

10.	Larynx (Supraglottic)	T4AN0	55/20fr	L	Epiglottis, vocal cord	11	Infield	-

11.	Larynx (Supraglottic)	T3N3	55/20fr	L, R	Stoma Left II, III, IV LN	24	Infield	Lung (Synchronous)

12.	Oropharynx	T4AN2C	55/20fr	L, R	Base of tongue, left tonsil Left level II, III, IV LN	13	Infield	-

13.	Larynx (supraglottic)	T3N2C	66/33fr	L	Supraglottic area	16	Infield	-

14.	Hypopharynx	T4AN0	55/20fr	L	Post hypopharyngeal wall extending upper oesophageous	16	Infield	-

**Induction chemotherapy + chemoradioherapy**								

15.	Larynx (Supraglottic)	T4AN2C	65/30fr	R	Right level II-III LN	12	Infield	Bone (Synchronous)

16.	Hypopharynx	T2N3	65/30fr	L, R	Postcricoid tumor with extensive nodal spread	20	Marginal	Lung (Synchronous)

17.	Oropharynx	T4AN2B	68/34fr	R	Left level III-IV LN	22	Infield	-

18.	Oropharynx	T3N3	70/35fr	L, R	Supraglottic region, inferior aryepiglottic fold Level IV LN	18	Infield	Lung (Synchronous)

19.	Oropharynx	T4AN2C	68/34fr	L	Right post parapharyngeal	23	Outfield	-

20.	Larynx (Supraglottic)	T4N0	55/20fr	L	Right pyriform fossa	21	Infield	-

Abbrevations: L: local recurrence; R: regional recurrence; NA: not applicable; XRT: radiotherapy; LN: lymph node; fr: fraction

6 of the total of 20 cases of LRR occurred in the group of 8 patients who had had a less than CR to non-surgical treatment and had subsequently undergone salvage surgery. In 5 of these 6 cases recurrence was in-field, and one was a marginal recurrence. All 3 of the patients who underwent a salvage laryngectomy developed an in-field stomal recurrence (patients 8,9,11). Among the patients with isolated in-field recurrences, 3 patients had undergone a right radical neck dissection prior to radiotherapy, and 2 of these patients recurred in right neck with synchronous DM (patients 3,15). Although the majority of treatments were bilateral, unilateral treatment was delivered in 9 patients based upon tumour stage, prior neck dissections or patient factors. However, only two out-of-field recurrences were in the untreated contralateral neck. These two patients also had simultaneous DM. In addition, 2 out-of-field regional relapses were associated with omission of high risk elective lymph node regions from CTV at the time of radiotherapy planning. In an 87 years old patient with T2N1 oral cavity tumour, the radiotherapy field was limited to the right upper neck because of age and co-morbidities (patient 5, Figure 1). The anterior neck and level VI lymph node regions were not included in CTV in a case with a T4AN0 glottic larynx (patient 2). The single case of an outfield local recurrence occurred in a patient with T4AN2C oropharyngeal tumor (patient 19); this patient had not been treated with standard radiotherapy techniques and had a high field match with the lower neck field in order to spare the larynx.

### Prognostic factors

On univariate analysis of all patients age, T stage, and tumour subsite were examined for their relationship with PFS and CSS (Table 4). Patients older than 60 years had significantly lower PFS (p = 0.013) and CSS (p = 0.002) rates. In addition, CSS (p = 0.0003) and PFS (p = 0.0002) rates were significantly higher in patients with T1-T2 disease compared with T3-4 disease. Both the PFS and CSS rates were significantly better for patients with oropharyngeal tumour compared to non-oropharyngeal tumours (p = 0.002 and p = 0.001 respectively). Nodal stage and the use of chemotherapy were not statistically significant prognostic factors (Table 4). Variables evaluated by multivariate analysis included age, tumour subsite and T-stage. T-stage was found to be a significant independent factor affecting both PFS and CSS rates (p = 0.006 and p = 0.008 respectively). Age and tumour subsite were also independent prognostic factors with a limited significance for CSS rate (p = 0.05) (Table 5).

**Table 4:** Univariate analysis of progression-free survival and cause-specific survival in all patients.

		PROGRESSION-FREE SURVIVAL		CAUSE-SPECIFIC SURVIVAL	
**Factors**	**N**	**3 years (%)**	**P**	**3 years (%)**	**P**

**Age group**					
≤60 years	83	75.8	**0.013**	75.9	**0.002**
>60 years	68	57		55.6	

**Tumour subsite**					
Oropharyngeal tumours	81	80.4	**0.002**	79.5	**0.001**
Non-oropharyngeal tumours	70	52		51.6	

**T stage**					
Tx, T1, T2	52	86.7	**0.0002**	87.6	**0.0003**
T3-T4	99	57.9		56.1	

**Nodal Status**					
N0-N1	60	73.6	0.395	70.7	0.369
N2-3	91	64.1		56.1	

**Treatment types**					
With Chemotherapy	115	70.4	0.409	69	0.266
Without chemotherapy	36	58.2		59.5	

**Table 5:** Multivariate analysis of prognostic factors for progression-free survival and cause-specific survival.

	PROGRESSION-FREE SURVIVAL		CAUSE-SPECIFIC SURVIVAL	
	
	P-value	Hazard ratio (95% CI)	P-value	Hazard ratio (95% CI)
**Age**	0.117		0.05	
**≤60 years**		0.658 (0.362-1.197)		0.559 (0.312-1.001)
**>60 years**		1		1

**T stage**	0.006		0.008	
**Tx, T1-T2**		0.288 (0.12-0.694)		0.332 (0.146-0.753)
**T3-T4**		1		1

**Tumour subsite**	0.06		0.05	
**Oropharyngeal tumours**		0.551 (0.297-1.021)		0.554 (0.305-1.005)
**Non-oropharyngeal tumours**		1		1

Abbrevations: CI: Confidence interval

## Discussion

A critical aspect of the management of HNSCC is to understand the patterns of treatment failure, in order to guide future attempts to optimize radiotherapy planning and improve the therapeutic ratio. If treatment failures are predominantly distant, intensification of systemic therapy may be needed to improve outcomes. By contrast, if failures are loco-regional, this may emphasize the need to identify patients with a radiation resistant tumour subpopulation and underlie the rationale for studies of dose escalation to the highest risk regions.

In this study, 82.8% of the patients achieved a complete tumour response 4 months after completion of therapy. The 4 month timepoint used here is intended to allow adequate time for the response to radiotherapy. Pacagnelli et al. showed that an 8 week response assessment is too early, with more complete responses being seen at 8 months than 8 weeks post-treatment [10]. We have demonstrated that 3-year local and regional control was high in these patients with rates of 86.8% and 89.5%, respectively. In 125 complete responders, there were 20 LRR. Amongst the 117 patients who achieved a CR to (chemo)-radiotherapy without salvage surgery, there were only 14 LRR. Six of the 8 patients who had undergone salvage surgery after a failure to achieve a complete response had a locoregional recurrence and 5 of these cases failure was infield. One third of cases of LRR were associated with the development of synchronous DM. DM in the absence of LRR was uncommon, occurring in only 9 of 125 patients.

Analysis of patterns of failure in relation to the dosimetry of the radiotherapy plan, demonstrated that 76% of LRR occurred within the PTV (12 of 14 local recurrences and 7 of 11 regional recurrences). There were no cases of isolated recurrences within the volume treated by the lower dose prophylactic anterior neck fields. 12 of the patients with LRR had been treated with hypofractionated 55Gy in 20 fractions. Based upon this data, and national guidance [11], hypofractionated treatment for locally advanced HNSCC has been abandoned in our centre. Five patients had an out-of-field LRR. Four of them were treated with radical radiotherapy only. Two were found to have simultaneous out-of-field recurrences and DM. Out-of-field relapse was mainly associated with deliberate omissions of elective lymph node regions at the time of radiotherapy planning. This omission of elective nodal regions reflects the nature of the patient population with locally advanced HNSCC, in which treatment has to be individualized on the basis of age, comorbidity, treated volume, disease extent. A marginal recurrence at the edge of the PTV was seen in only one patient.

Uncertainty in the demarcation of the target volumes is one of the major limitations to improving outcomes of HNSCC with radiotherapy. The extent of gross tumour determined clinically and using the planning CT scans is not straightforward. A major difficulty is the delineation of lymph node groups at risk of subclinical disease. It has been shown that there is significant variation in nodal volumes when defined by different radiation oncology specialists [12,13]. Our data, demonstrating that LRR are predominantly occurring in-field, is reassuring in terms of the quality of target volume definition and elective nodal targets.

There is limited published literature regarding patterns of disease recurrence after radiotherapy for HNSCC. This has been best documented in several IMRT series. Recently the IMRT technique has replaced conformal 3D-planning in many centres. It is important to document the recurrence patterns of IMRT series which may carry a higher risk of geographical tumor misses and compare with 3-D conformal techniques. Most IMRT series have focused on decreased rates of xerostomia in HNSCC with IMRT or investigated the pattern of failure in patients treated with surgery and/or chemotherapy in addition to IMRT [14-16]. In one of the larger series, Chao et al. analyzed 126 head and neck cancer patients treated with IMRT delivered with radical intent without surgery in 41% of patients, and post-operatively in 59% of patients [17]. After a median follow up of 26 months, 17 LRR were noted, of which 9 were in-field, 3 were marginal failures and 5 were outside of the IMRT field. Eisbruch et al. reported results of 133 patients treated with parotid-sparing 3D-CRT or IMRT and of the 21 LRR, 17 were in-field and 4 were marginal [18]. Studer et al. reported a 80% and 87% local and regional control rate, respectively and 95% of failures occurred in-field following IMRT [19]. Therefore, the majority of failures are in-field in reported IMRT series. However, given the heterogeneity of disease sites, stage, treatment types and the number of patients in these studies, it is not possible to accurately compare the rate of recurrence in our patients to patients treated with IMRT. In addition, longer follow-up is needed to validate IMRT findings, particularly with regards to LRC and late complications.

The use of 3D-CRT radiotherapy allowing increased GTV doses within normal tissue tolerances, and the increasing utilization of combined chemotherapy with radiotherapy, is likely to lead increasing rates of LRC. This may alter failure patterns on HNSCC. Indeed, some studies have shown that DM have become an increasingly important site of recurrence and mortality [20,21]. In our series, the 3-year DMFS rate was 87.3%. By contrast, 26 of 151 patients never achieved LRC, and a further 20 of 125 complete responders subsequently developed LRR. These data highlight the importance of continued efforts to improve therapy to enhance LRC; the large majority of treatment failures remain locoregional failures.

HNSCC are heterogenous in their aetiology and behaviour. HPV DNA has been found in approximately 25% of HNSCC and HPV-associated tumours tend to arise in oropharynx but not in the larynx [22]. In addition, HPV-positive tumours are associated with a better prognosis [23]. This may explain better treatment results in oropharyngeal tumours in our study. In addition, patients with tumour stage T3-T4 had a higher risk of PFS and CSS both with univariate and multivariate analysis. However, we did not observe a statistically significant effect of N-stage. Hence, our series suggests that T-stage is the predominant prognostic factor. Accurate stratification of patients in terms of prognosis is likely to be important in identifying subgroups that may benefit from an intensification of treatment.

Although this is a series in a single cancer centre, under the supervision of a dedicated head and neck oncology team, the treatment related death rate was 4%. The most common cause of death was aspiration pneumonia. However, co-morbidities, limited performance status, poor social support and heavy alchohol consumption may have had an impact upon toxicity (Table 6). Based upon these data, we now routinely offer prophylactic gastrostomy-tube placement for patients receiving concurrent chemoradiotherapy or have heavy alcohol consumption, and poor social support. We believe that better patient selection and provision of intensive, experienced, multidisciplinary support during treatment and beyond, decreases the mortality rate.

**Table 6:** Characteristics of patients who were died due to treatment toxicity

**Pat. no**.	Age	Diagnosis	Stage	Treatment type	Given XRT dose (Gy/fr)	Smoking/Alcohol	Cause of death	Comorbidities
1	69	Hypopharynx	III	XRT	30.25/11fr	Heavy/heavy	Aspiration Pneumonia, MRSA	COPD, 1994 Lung Ca. lobectomy

2	57	Oropharynx	IVB	Induct chemox3+XRT	35.75/13fr	Ex/Heavy	Aspiration Pneumonia	AF, DM

3	65	Larynx (Glottic)	III	XRT	55/20fr	Ex/Heavy	Aspiration Pneumonia, MRSA	COPD, Asthma, old tuberculosis

4	64	Hypopharynx	IVB	Induct Chemox3+XRT	46/23fr	Heavy/heavy	Aspiration Pneumonia	-

5	84	Larynx (Supraglottic)	IVB	XRT	55/20fr	Social	Aspiration Pneumonia	Cardiac problems

6	61	Oropharynx	IVA	Concomitant chemoXRT	55/20fr	Heavy/heavy	Neutropenia, emergency admission refuse NG	-

*NG tube feeding was inserted to patients' number 1-4, when the complication was seen. None of the patients had prophylactic PEG or NG. Abbrevations: Chemo: chemotherapy; XRT: radiotherapy; COPD: chronic obstructive pulmonary disease; MRSA: methicilline resistant staph aerous

In summary, this series demonstrates that conformal (chemo-)radiotherapy offers high rates of LRC and OS. Systemic targeting may improve outcomes, however the majority of LRR after a CR to treatment occur within the PTV and isolated distant metastatic recurrence was uncommon. This may relate to intrinsic radioresistance or factors such as tumour hypoxia. These data provide a clear rationale for efforts aimed at improving locoregional tumour control. Useful approaches may include induction chemotherapy regimens, biological therapies, radiosensitisers, altered fractionation, and dose escalation. It is likely that the future will involve the identification of predictive markers of treatment response, identifying patients likely to fail locoregionally. This may allow the selection of an individually tailored treatment regimen.

## Competing interests

The authors declare that they have no competing interests.

This study was presented in part at the 51^th ^Annual Meeting of the American Society of Therapeutic Radiology and Oncology, Chicago, November 1-5,2009

## Authors' contributions

DCO: Data collection, analysis, interpretation, manuscript preparation and approval; RJDP: Data interpretation, manuscript preparation and approval; BC: Carried out the radiological data analysis, interpretation, manuscript approval; SW: Carried out the dosimetric analysis, interpretation, manuscript approval; MSS: Performed the statistical analysis, manuscript approval; AC: Data collection, manuscript approval; KD: Original Concept, Manuscript approval; CC: Original Concept, Manuscript approval; MS: Original concept, data interpretation, manuscript approval.

All authors read and approved the final manuscript.
